# How special is the biochemical function of native proteins?

**DOI:** 10.12688/f1000research.7374.1

**Published:** 2016-02-23

**Authors:** Jeffrey Skolnick, Mu Gao, Hongyi Zhou

**Affiliations:** 1 Center for the Study of Systems Biology, School of Biology, Georgia Institute of Technology, Atlanta, GA, USA

**Keywords:** Protein structure, protein interactions, protein conformation, biochemical function, native proteins, Enzymatic active sites

## Abstract

Native proteins perform an amazing variety of biochemical functions, including enzymatic catalysis, and can engage in protein-protein and protein-DNA interactions that are essential for life. A key question is how special are these functional properties of proteins. Are they extremely rare, or are they an intrinsic feature? Comparison to the properties of compact conformations of artificially generated compact protein structures selected for thermodynamic stability but not any type of function, the artificial (ART) protein library, demonstrates that a remarkable number of the properties of native-like proteins are recapitulated. These include the complete set of small molecule ligand-binding pockets and most protein-protein interfaces. ART structures are predicted to be capable of weakly binding metabolites and cover a significant fraction of metabolic pathways, with the most enriched pathways including ancient ones such as glycolysis. Native-like active sites are also found in ART proteins. A small fraction of ART proteins are predicted to have strong protein-protein and protein-DNA interactions. Overall, it appears that biochemical function is an intrinsic feature of proteins which nature has significantly optimized during evolution. These studies raise questions as to the relative roles of specificity and promiscuity in the biochemical function and control of cells that need investigation.

## Introduction

Often proteins adopt a unique, thermodynamically stable native conformation that can perform an amazing variety of biochemical functions ranging from enzyme catalysis and signal transduction to force generation ^1^. When one looks at the diversity of protein functions, one cannot but wonder how they came about. At first glance, the natural tendency is to assume that their remarkable properties mainly arise from evolutionary selection, with the inherent background features that reflect the physical properties of proteins playing a minor role. If so, proteins should exhibit little intrinsic background function, and those that do should be very rare ^2– 8^. The fundamental problem with this viewpoint is that for selection to occur, there must be some background function on which to select; in practice, low-level function emerges remarkably quickly in function design studies ^9– 11^. The key issue is how to estimate this random background probability for function. Here, computer experiments can provide important insights ^12– 16^. For function to occur, often there must be an interaction between molecules. Thus, in what follows, we examine the inherent ability of proteins to engage in small molecule protein-protein and protein-DNA interactions. Surprisingly many biochemical properties of native proteins are found in a library of stable artificial structures generated without any selection for biochemical function. Remarkably, this includes enzymatic active sites and, at much lower frequency, pockets that loosely resemble the enzymatic binding pocket. This suggests that functional selection by evolution is most likely involved in fine-tuning rather than in generation of intrinsic function. If so, marginally stable proteins are inherently ready to engage at low level in the biochemical functions necessary for life.

## Generation of an artificial protein library to examine their intrinsic functional features

To separate out the intrinsic properties of proteins from those due to evolution, one could design proteins without selection for function, solve their structures, assay their function, and explore their similarity to native proteins ^17– 19^. To cover all representative protein functions would be a long, expensive process that is, at present, impractical. Rather, we chose to perform a series of computer experiments ^12– 16^, where a library of compact homopolypeptides from 40–250 residues in length were generated using the TASSER structure prediction algorithm ^20^. Then, sequences with protein-like composition were selected by optimizing their thermodynamic stability in the putative fold of interest ^13^. These artificial proteins are termed the “ART” protein library.

## Small molecule ligand-binding pockets

Having the ART library in hand, we compared the small molecule ligand-binding pockets to those in native proteins. Remarkably, all ligand-binding pockets in native proteins have a statistically significant match to the pockets in the ART library. This suggests that the library of all ligand-binding pockets, the “pocketome” ^21^, is likely complete and arises from defects in packing of compact secondary structures, as proteins without secondary structure have tiny pockets that cannot bind biologically relevant molecules ^22^. In practice, for single-domain globular proteins, the space of protein pockets is covered by a remarkably small number (about 500) of representative pockets. These results are consistent with a large-scale study on a non-redundant set of ~20,000 known ligand-binding pockets that finds their structural space is crowded, likely complete, and represented by a similar number of pockets ^23^. Similar protein pockets occur in proteins that have globally unrelated folds. On the other hand, closely related proteins need not have similar pockets. The presence of similar pockets capable of binding similar, if not identical, ligands in multiple protein families rationalizes at least part of the reason why drugs have unintended side effects.

## Ability of ART proteins to bind small molecule metabolites

A representative set of 1400 Kyoto Encyclopedia of Genes and Genomes (KEGG) molecules (clustered using Tanimoto coefficient TC=0.7 from a total 12,271 molecules ^24^) were screened against a representative set of ART proteins using the FINDSITE ^comb^ virtual ligand screening algorithm ^25^. FINDSITE ^comb^ has an average success rate of 21% at identifying micromolar or better binders when 50 or fewer small molecules are screened ^26^. Enrichment factors of the top 1% of ranked ligands relative to a set of 69,271 background molecules (the ZINC 8 library ^27^) culled with a TC ^28^ of 0.7 were 2.57, with 98.6% of ligands having an enrichment factor >1 (the random background result). We found that the median number of binding targets per KEGG molecule is 35, quite close to the number (38) of proteins predicted to bind to drugs in the human exome ^29^. Of these 1400 molecules, 1186 or 84.7% molecules have at least one binding target, and the median number of small molecules that bind per protein is 36 (as compared to 57 drugs per protein, but this discrepancy may be due to the small number of metabolites considered).

We next explored the enrichment factor of metabolites predicted to bind to proteins in a given metabolic pathway. We define the enrichment factor of a pathway as

$$E_{p}=\frac{{}_{molecules\;in\;pathway}\;number\;of\;binding\;protein\;targets}{{}_{molecules\;in\;pathway}\;number\;of\;protein\;targets\;by\;random\;selection}$$

The average enrichment factor of 238 KEEG pathways is 14.6 with 84.0% of pathways having an *E _p_* >1. Thus, there is a significant tendency for metabolites in existing pathways to bind to ART proteins even without any functional selection. As shown in Table 1, the top 18 most enriched pathways by FINDSITE ^comb^ include ancient pathways associated with glycolysis ^30^, the metabolism of ancient amino acids alanine, aspartate, and glutamate ^31, 32^, and glycerolipid metabolism ^33^. Thus, a subset of the top 18 pathways is believed to be ancient. However, the ability to bind a molecule is a necessary but insufficient condition for enzymatic activity, an issue we turn to next.

**Table 1.** Top 18 most enriched pathways by FINDSITE ^comb^.

Pathway	Enrichment factor	Number of proteins in the pathway
Insulin signaling pathway	77.5	67
Alcoholism	68.0	5
Amphetamine addiction	68.0	46
Cocaine addiction	68.0	42
Huntington's disease	68.0	36
Amyotrophic lateral sclerosis (ALS)	68.0	29
GABAergic synapse	68.0	28
Taurine and hypotaurine metabolism	68.0	27
Proximal tubule bicarbonate reclamation	67.3	14
HMG-CoA reductase inhibitors	59.8	4
Galactose metabolism	56.8	20
Phosphotransferase system (PTS)	54.1	n.a.
Glycerolipid metabolism	48.7	20
Butirosin and neomycin biosynthesis	45.9	3
Glyoxylate and dicarboxylate metabolism	45.9	18
Alanine, aspartate, and glutamate metabolism	45.6	27
Glycolysis/gluconeogenesis	39.7	30
Retrograde endocannabinoid signaling	39.6	23

## Enzymatic active sites

**Figure 1.**
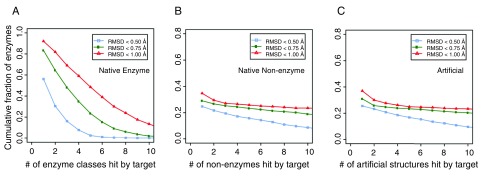
Cumulative fraction of enzymes whose active sites match pocket residues in ( **A**) other classes of enzymes in native structures with different first two digit Enzyme Commission (EC) numbers, ( **B**) in non-enzymes, and ( **C**) in ART structures. For each target enzyme, we count the number of alternative enzyme classes that contain at least a hit by the target enzyme at various root-mean-square-deviation (RMSD) cut-offs.

We next explored how special the active sites in enzymes are. To address this question, we undertook a large-scale search for amino acids with similar geometry and same residue identity as in enzyme active sites found in a manually curated set from the Catalytic Site Atlas (CSA) database ^34^. There, each entry corresponds to a protein chain with an experimentally determined structure in the Protein Data Bank (PDB) ^35^. In total, we studied 1373 protein chains that are annotated as being enzymes. For each target enzyme, we first detected pockets using a geometry-based method ^36^. We then scanned these pockets against known active sites of the template library of enzymes ^37^. If the target had an amino acid arrangement with a similar geometry as the active sites of a template enzyme whose root-mean-square-deviation (RMSD) from that of the known enzyme’s active site <1 Å RMSD and had 100% sequence identity, we considered it a hit. About 94% of the enzymes hit at least one template enzyme that had different first two-digit Enzyme Commission (EC) numbers, i.e. they are from very different enzyme classes. We further counted hits according to their enzyme classes at the four-digit EC level using various RMSD cutoffs ( Figure 1); 75% of target enzymes hit three or more enzyme classes below an RMSD of 1 Å, 54% below a RMSD of 0.75 Å, and 21% below a RMSD of 0.5 Å. Thus, in native proteins, the active sites of enzymes are not as rare nor as geometrically and chemically unique as previously thought; no more than 5000 or so ART structures were searched here.

**Figure 2.**
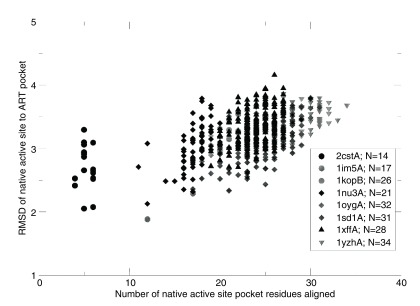
Root-mean-square-deviation (RMSD) of native enzymatic to ART pockets versus the number of pocket residues aligned. The number of residues in the native active site pocket, N, is shown in the figure legend.

Next, we performed a search of enzyme-like active sites in native structures of non-enzymes ( Figure 1B) and in the ART library ( Figure 1C). From a set of 4609 non-enzymes ^23^ and a set of the same number of randomly selected artificial structures, we first identified the largest pocket in these structures, then searched in these pockets for residues that resemble active sites in native enzymes. We only considered hits that had a different global structure with a template modeling (TM)-score <0.4 ^38^ (a threshold for structural significance) from a target native enzyme. Using the same criteria, at an RMSD <1 Å and 100% coverage and sequence identity, we found at least a hit for 35% of enzyme active sites in non-enzymes and a comparable value (37%) in artificial structures. For an RMSD <0.75 Å, 29% and 31% of native active sites were matched, respectively. Finally, at an RMSD <0.50 Å, 25% and 26% of native active sites were found for non-enzymes and artificial structures, respectively. Small-size active sites were mostly easy to find a hit: about 88% of three-residue active sites, 35% of four-residue active sites, and 0.3% of five-residue active sites were found in artificial structures. About 25% of enzymes had more than four hits in artificial structures. However, it should be pointed out that the global pockets in these matches usually did not have a significant similarity score to the native active site pocket, despite the high structural similarity of their active site residues. Whether these native non-enzymes could weakly catalyze a similar reaction in a different substrate is unknown, as there are other factors that could dictate enzymatic activity ^39^. To further investigate this issue, we froze the catalytic residues in the artificial structure of interest and generated sets of stable sequences for the given fold. We then examined whether artificial pockets globally similar to the active pocket in that native enzyme are generated. As shown in Table 2, depending on the particular ART structure, the success rates (p-values of the pockets <0.05) ranged from 0% to 1.5% of the sequences generated. Given a fixed orientation of the active site residues, there are certain backbone geometries that cannot accommodate the native pocket geometry in certain global folds. Consider, for example, a long narrow pocket. Given the location of the active site residues, it might have to penetrate the backbone for the pocket to be completely recapitulated; clearly, in such a situation, that native enzymatic pocket cannot occur. For successful cases, all of which have a globally unrelated fold to the native structure as assessed by their TM-score ^40^, one need only sample on the order of ~10 ^4^–10 ^5^ random sequences to generate a pocket that is at least weakly related to the native pocket. For these, the RMSDs of the aligned residues versus the number of aligned pocket residues for eight pairs of native enzymes-ART proteins are shown in Figure 2. The range of RMSD values is 2–4 Å and spans 4-35 residues. These pockets have p-values <0.05 associated with the pocket similarity (PS)-score ^37^. At this range of PS-scores ^23^, about 13% of ligands share significant chemical similarity as assessed by their TC ^28^.

**Table 2.** For a subset of ART proteins containing active site residues and geometrics, % of pockets that have a significant p-value to the native enzymatic pocket.

Native enzyme	Enzyme Commission (EC) number	% (number) of ART structures whose active site pocket has a p-value <0.05	Number of residues in the native pocket	Template modeling (TM)-score of ART template to native ^39^
2a8yB-ART1 ^a^	2.4.2.28	0% (0/11,680)	25	0.25
2cstA-ART1	2.6.1.1	0.33% (39/11,680)	14	0.23
2fniA-ART1 ^a^	2.6.1.92	0 (0/11,680)	45	0.25
1im5A-ART1	3.5.1.19	0.027% (3/11,520)	17	0.27
1kopB-ART1	4.2.1.1	0.21% (24/11,680)	26	0.28
1nu3A-ART1	3.3.2.8	0.38% (82/211,440)	21	0.27
1oygA-ART1 ^a^	2.4.1.10	0.0% (4/11,680)	32	0.23
1oygA-ART2	2.4.1.10	0.043% (5/11,680)	32	0.17
1sd1A-ART1	2.4.2.28	1.4% (167/11,680)	31	0.23
1sd1A-ART2 ^a^	2.4.2.28	0 (0/11,520)	31	0.27
1w23B-ART1 ^b^	2.6.1.52	0% (0/221,280)	10	0.19
1xffA-ART1	2.6.1.16	1.8% (211/11,680)	28	0.34
1yxhA-ART1	3.1.1.4	0.45% (52/11,520)	34	0.35
2z2xA-ART1	3.4.21.62	0% (0/11,680)	27	0.26

^a^No pockets matched even without the active site residue matching restraint imposed. ^b^8/221,280 pockets match without the active site residue matching restraint imposed.

## ART protein-protein and protein-DNA complexes

**Figure 3.**
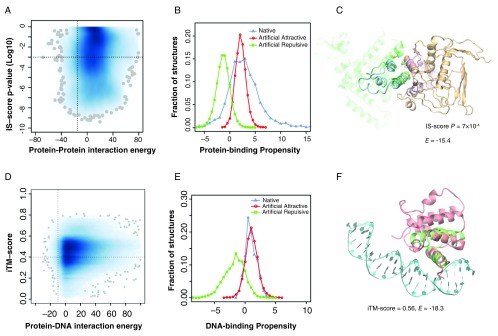
Artificial protein-protein, protein-DNA complexes. ( **A**) Statistics of putative artificial protein-protein complexes. Joint probability density of interaction energy *E _PP_* ^43^ and the p-value of the interface similarity (IS)-score ^42, 44^ between an artificial complex and its corresponding native template. Darker blue indicates higher density, with the 100 lowest density spots represented by grey spheres. A vertical/horizontal dashed line is placed at *E _PP_* = -15 (a cut-off for high likelihood of interaction) and P = 1×10 ^-3^. ( **B**) Protein-binding propensity scores (>0 implies favorable binding) of native protein-protein interfaces versus putatively attractive ( *E _PP_* <-15) and repulsive ( *E _PP_* >10) artificial protein-protein interfaces. ( **C**) Example of an ART protein-protein complex. The complex was built by superimposing two artificial structures (cyan and orange) onto a native dimeric template (Protein Data Bank [PDB] code 2f4m, chain A and B, colored in green and purple). Interface alignment according to iAlign ^42^. Both structures are shown in line representations, with the non-interfacial regions of the native template shown in transparent mode for clarity. ( **D**) Statistics of artificial DNA-protein complexes. Joint probability density of DNA-protein interaction energy, *E _DP_* ^46^, and the interfacial template modeling (TM)-score ^22^ between an ART protein and its corresponding native template. A vertical/horizontal dashed line is placed at *E _DP_* = -10 and *i*TM-score = 0.4. ( **E**) DNA-binding propensity scores (>0 implies favorable binding) of native DNA-protein interfaces versus putatively attractive ( *E _DP_* <-10) and repulsive ( *E _DP_* >10) artificial DNA-protein interfaces. ( **F**) Example of an artificial DNA-protein complex. The complex was built by superimposing the ART structure (red) onto a native template (PDB code 1akh, the native protein and DNA are colored in green and cyan, respectively).

Not only do the ART structures resemble native proteins in terms of fold similarity and ligand-binding pocket but docked ART structures match native protein-protein interfaces, suggesting that the space of protein-protein interfaces is complete and covered by roughly 1000 distinct types of interfaces ^15^. Interestingly, they also possess the ability to form native-like protein-protein and protein-DNA complexes. To demonstrate this, we randomly selected 30,000 pairs of ART structures in representative native-like folds; each fold had 80 protein-like sequences predicted to be stable for that fold. This gives 192 million pairs of ART monomers. To find possible native-like complexes, a simple yet efficient strategy was adopted. First, we compared the backbone structural similarity of ART monomeric structures with monomeric structures found in a library of 1690 non-redundant native dimeric complexes ^41, 42^. Using structural alignments, we built putative complexes by superimposing individual ART structures onto their corresponding aligned monomers from the native templates ^38^. We only considered those putative complexes that had significant global structural similarity and were aligned to more than 50% of the native interface. This yielded 135,942 putative ART complexes, and each had a corresponding native protein complex as its template. As shown in Figure 3A, the vast majority were either energetically unfavorable or did not share significant structural similarity to their corresponding template. However, about 2584 ART monomer pairs, or 1.3×10 ^-5^ of the total, had strongly favorable interactions and shared significant structural similarity with their templates. These ART complexes may be considered native-like. In general, attractive ART interfaces are enriched in hydrophobic residues. The protein-binding propensity scores of attractive ART complexes overlap with the scores of native complexes ( Figure 3B). An example is illustrated in Figure 3C. This ART complex has a favorable interaction energy of -15.4 ^43^ and shares significant interface similarity (IS) at an IS-score p-value of 7×10 ^-4^ with respect to the closest native protein complex ^42, 44^. Thus, putative native-like protein-protein complexes are found without any selection whatsoever for protein-protein interactions.

Similarly, we searched for ART structures with a strong native-like DNA-binding propensity. A set of 32,279 ART folds, each with 80 sequences selected for stability, was scanned. As above, we first performed all-against-all structural comparison between individual ART structures and native protein structures found in 1350 experimentally determined protein/DNA complexes ^45^. The vast majority had either energetically unfavorable DNA-protein interfaces or did not share significant structural similarity with their corresponding native protein templates ( Figure 3D). However, 2515 ART proteins, or 9.7×10 ^-4^ of the total, had strongly favorable interactions and significant structural similarity to DNA-binding templates. These ART proteins may be considered to have native-like DNA-binding function. Analysis of their DNA-binding interface suggests that they have a large number of positively charged Arg and Lys residues, especially Arg, which is enriched at the DNA-binding interface. This is reasonable, as DNA molecules are negatively charged ^46^. By comparison, DNA-repulsive ART interfaces have a similar sequence composition as native non-DNA-binding surface residues. The DNA-binding propensity scores of DNA-attractive ART structures overlap with the scores of native DNA-binding proteins ( Figure 3E); an example is displayed in Figure 3F. Thus, intermolecular interactions between proteins or involving DNA and proteins could emerge without any selection.

## Conclusion

Comparison of the properties of native proteins with those of ART structures selected for stability, but not function, shows that many of the properties seen in native proteins emerge as intrinsic features resulting from the packing of secondary structures. The space of small molecule ligand-binding sites found in native and artificial protein structures is shown to be complete, with about 500 representative pockets. Similarly, pockets can occur in proteins with different global folds, while dissimilar pockets are found in proteins that are closely related by evolution with similar structures. Thus, the geometry and amino acid composition of protein pockets are only weakly coupled to the global fold of a protein. The likelihood that a given small molecule differentially interacts with multiple proteins in different families is high. How nature gets around this promiscuity to generate and control cells is a key unanswered question. If cells operated on the basis of one small molecule-one protein target, it is easy to understand how the organized biochemical processes of life occur, but this is apparently not the case ^29^. In practice, the situation is possibly more complex.

Remarkably, ART proteins are predicted to bind weakly to a sufficient number of native metabolites that metabolic pathways are enriched relative to what would be expected at random. Moreover, the ART library has significant matches to the active sites and their associated pockets of enzymes in native proteins (which also are found in putative non-enzyme native proteins). Thus, active site geometry is not special, and it appears that a significant fraction of the biochemistry of life, at least at very low level, is encoded in the physical properties of proteins. If this view is true, and these observations need to be experimentally validated, this has significant implications for the origin of life.

Turning to the likelihood of protein-protein and protein-DNA interactions occurring at random, the strong implication is that a tiny fraction of proteins can engage in at least intermolecular interactions without functional selection. Once again, intermolecular interactions emerge as an inherent feature of proteins due to the packing of secondary structures ^22^. Again, there is the implication of weak omnipresent promiscuous interactions in a cell. How cells sort out the myriad of weak interactions relative to the small fraction of specific ones needs to be better clarified. Part of the answer may lie in subcellular localization.

Overall, these studies suggest that the “special” functional properties of proteins are not as special as commonly viewed. Pockets, enzymatic active sites, and native-like protein-protein and protein-DNA interactions are found in artificial protein structures that are selected for stability and nothing more. The packing of secondary structure is found to provide the geometric context for pockets and intermolecular interfaces. The requirements that a protein be compact and water soluble and adopt a thermodynamically unique conformation give rise to protein sequences that recapitulate the necessary functional features (at least at low level) of real native proteins. Overall, it appears that biochemical function is merely an intrinsic feature of proteins that nature has then significantly optimized.
